# Genome-Wide Scan for Runs of Homozygosity Identifies Candidate Genes Related to Economically Important Traits in Chinese Merino

**DOI:** 10.3390/ani10030524

**Published:** 2020-03-20

**Authors:** Sangang He, Jiang Di, Bing Han, Lei Chen, Mingjun Liu, Wenrong Li

**Affiliations:** 1 Key Laboratory of Ruminant Genetics, Breeding & Reproduction, Ministry of Agriculture; Key Laboratory of Animal Biotechnology of Xinjiang, Institute of Biotechnology, Xinjiang Academy of Animal Science, Xinjiang, Urumqi 830026, China; hesangang@xjaas.net (S.H.); bingh_xj@sina.com (B.H.); chenlei0991@126.com (L.C.); xjlmj2006@126.com (M.L.); 2 Xinjiang Wool sheep and Cashmere Goat Key Breeding Lab, Institute of Animal Science, Xinjiang Academy of Animal Sciences, Xinjiang, Urumqi 830026, China; dijiang69@163.com

**Keywords:** runs of homozygosity, Chinese Merino sheep, candidate genes, genomic inbreeding coefficient

## Abstract

**Simple Summary:**

Runs of homozygosity (ROH) are commonly used to estimate inbreeding coefficients and identify selection signatures in livestock population. The present study determined ROH patterns, estimated the inbreeding levels, and identified the genome regions with high ROH frequency (ROH hotspots) in Chinese Merino. Our results showed that the genome of Chinese Merino harbored lower ROH abundance. Moreover, the inbreeding levels were relatively low. Thirteen ROH hotspots consisting of 190 genes were identified. The ROH hotspots overlapped the selective signatures might be associated with body size, horn traits, immune traits and environment adaption. These findings could contribute to an optimum breeding program by identifying the candidate genes related to economically traits in Chinese Merino.

**Abstract:**

In this study, we estimated the number, length, and frequency of runs of homozygosity (ROH) in 635 Chinese Merino and identified genomic regions with high ROH frequency using the OvineSNP50 whole-genome genotyping array. A total of 6039 ROH exceeding 1 Mb were detected in 634 animals. The average number of ROH in each animal was 9.23 and the average length was 5.87 Mb. Most of the ROH were less than 10 Mb, accounting for 88.77% of the total number of detected ROH. In addition, *Ovies aries* chromosome (OAR) 21 and OAR3 exhibited the highest and lowest coverage of chromosomes by ROH, respectively. OAR1 displayed the highest number of ROH, while the lowest number of ROH was found on OAR24. An inbreeding coefficient of 0.023 was calculated from ROH greater than 1 Mb. Thirteen regions on chromosomes 1, 2, 3, 5, 6, 10, 11, and 16 were found to contain ROH hotspots. Within the genome regions of OAR6 and OAR11, *NCAPG/LCORL*, *FGF11* and *TP53* were identified as the candidate genes related to body size, while the genome region of OAR10 harbored *RXFP2* gene responsible for the horn trait. These findings indicate the adaptive to directional trait selection in Chinese Merino.

## 1. Introduction

Runs of homozygosity (ROH) are defined as contiguous homozygous genotype segments that arise from the transmission of identical haplotypes from parents to their offspring [1]. The distribution, frequency, and length of ROH are commonly associated with several factors, such as selective breeding, natural selection, recombination rate, and demographic history [2]. In animal breeding programs, to avoid inbreeding depression, a highly sensitive and accurate estimation of inbreeding is of great importance [3]. The classical method for calculating the inbreeding coefficient is through the use of pedigree data. However, the pedigree-based inbreeding coefficient (F_PED_) is largely dependent on the accuracy, completeness and depth of pedigree information, and pedigree errors are common in many livestock populations. Moreover, F_PED_ tends to ignore the history of selective breeding and underestimates the level of inbreeding, possibly due to the pedigree records [4].

At present, several alternative methods have been proposed to estimate inbreeding coefficient based on the whole-genome single nucleotide polymorphism (SNP)-derived metrics of autozygosity. For instance, the ROH-based inbreeding coefficient (F_ROH_) is an optimal approach to reflect the present-day and ancient inbreeding levels [5]. This method has been widely used to estimate the genomic inbreeding of many livestock populations in recent years [6,7,8,9,10]. The animals subjected to direct selection for target traits may form selection signatures or selective sweeps in the genome regions. With the loss of nucleotide diversity and increased homozygosity, the selected genome regions tend to generate ROH islands or ROH hotspots compared to the rest of the genome [11]. ROH hotspots are not randomly distributed across the genome and are shared by all individuals within a breed [12]. ROH are commonly used to identify genome regions under putative selection in order to map the candidate genes responsible for economically important traits in livestock populations [13,14,15,16,17,18,19,20].

Chinese Merino (Xinjiang type) is the most well-known sheep breed with fine wool in China. The breeding goals for a sheep breed include fine and soft wool, polled females, and large body size. However, there is limited information about the distribution of ROH across the genome in Chinese Merino. Therefore, this study aimed to investigate the characteristics of ROH in Chinese Merino using the Illumina Ovine 50 K SNP BeadChip, as well as to identify the genomic regions with high ROH frequency, namely ROH islands or ROH hotpots, which could have occurred during the selective breeding for production-related traits.

## 2. Materials and Methods

### 2.1. Sampling, Genotyping, and Data Quality Control

Ear tissue samples were collected from 635 female Chinese Merino (Xinjiang type) sheep in Bohu farm and Gongnaisi farm (Xinjiang, China). All samples were genotyped for 54241 SNPs using the OvineSNP50 Genotyping BeadChip (Illumina Inc., San Diego, CA, USA). The quality control of SNP data was performed with PLINK v1.90 software. SNPs were removed from the analysis if they did not align to the autosome of *Ovis aries*. The eligible SNPs were then filtered according to the following criteria: (1) Call frequency ⩾ 0.95, (2) minor allele frequency (MAF ⩾ 0.01), (3) Hardy–Weinberg equilibrium (HWE > 0.000001), and (4) individual call rate >0.9. The samples that did not satisfy these criteria were excluded.

### 2.2. Selection Criteria for Runs of Homozygosity

The runs of homozygosity (ROH) in each sample were evaluated using PLINK v1.90 software. The following criteria were used for ROH estimation: (1) The minimum length of ROH was set to 1 Mb, (2) five missing SNPs and up to one possible heterozygous genotype were permitted for each ROH window, (3) the minimum number of SNPs was set to 40, (4) a minimum density of one SNP every 100 kb, and (5) the maximum gap between two consecutive SNPs was set to 1 Mb.

### 2.3. Distribution of Runs of Homozygosity

The mean number, average length, and total number of ROH in each animal were estimated. The percentage of chromosomes covered by ROH was also determined. First, the mean ROH length was calculated by summing all ROH (Mb) on the *Ovies aries* chromosome (OAR) and then dividing by the number of animals that had ROH on that OAR. Subsequently, the mean ROH length was divided by the length (in Mb) of OAR.

### 2.4. Genomic Inbreeding Coefficients

Based on ROH data, the genomic inbreeding coefficient (F_ROH_) of each animal was calculated as follows: F_ROH_ = L_ROH_ / L_AUT_, where L_ROH_ is the total length of all ROH in an animal genome, and L_AUT_ refers to the length of autosomal genome covered by the SNPs included in the array.

In addition, the values of chromosomal F_ROH_ (F_ROHOAR_) were estimated as follows: F_ROHOAR_ = L_ROHOAR_ / L_OAR_, where L_ROHOAR_ is the total length of ROH in an OAR, and L_OAR_ is the length of an OAR covered by the corresponding SNPs.

### 2.5. Identification of Common Runs of Homozygosity

To identify the most commonly ROH-associated genomic regions, the percentage of SNP occurrence in ROH was calculated by counting the number of times each SNP within the detected ROH and then dividing the number of animals. The obtained data were plotted against the chromosomal positions of SNPs. The genomic regions with top 1% SNPs were considered as a potential ROH ROH hotspot in the genome [21,22].

To verify whether recombination can affect ROH hotspots, ROH were mapped based on the position of SNP (i.e., position in the genetic map of female sheep) [23]. The average recombination rate (cM/Mb) in each ROH hotspot was calculated. Next, the genetic map of ROH lengths was used to deduce demographic event using the method reported by Thompson et al. [18]. The methods used for meat sheep breeds [19] and Valle del Belice sheep [17] were adopted in this study, in which four similar ROH length categories were applied.

The identified genomic hotspots were used for gene annotation using Biomart according to the sheep reference genome (Oar 3.1). Gene ontology (GO) and Kyoto Encyclopedia of Genes and Genomes (KEGG) pathway enrichment analyses were then performed using Metascape with default parameters [24].

## 3. Results

### 3.1. Distribution of Runs of Homozygosity

**Figure 1:**
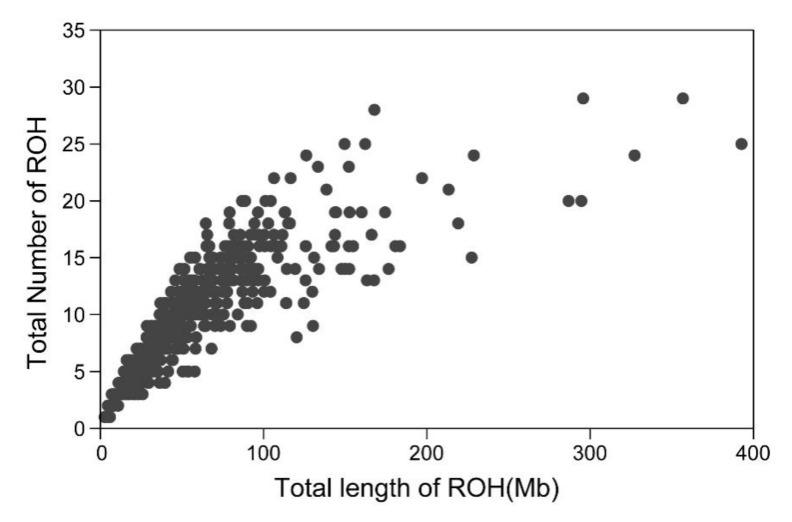
Total number of runs of homozygosity (ROH) exceeding 1 Mb and total length of genome (in Mb) covered by ROH segments in Chinese Merino.

In total, 6039 ROH with varying length were identified in all 635 samples, and the average length of ROH was 5.87 Mb. Notably, the longest ROH (up to 60.19 Mb) containing 1247 SNPs was found on chromosome 3, while the shortest ROH was only 1.54 Mb. Among the studied samples, 634 of them had at least one ROH. The average number of ROH in each animal was 9.23 (ranging from 1 to 29 ROH), while the average total length of ROH was 55.78 Mb. Three Merino sheep had the highest homozygosity levels of 392.74 Mb, 356.76 Mb and 327.21 Mb, which were relatively close to 15% of the genome. On the contrary, the Merino sheep with lowest homozygosity had only one ROH of 2.66 Mb. Figure 1 demonstrates the number and total length of ROH in Chinese Merino.

**Figure 2:**
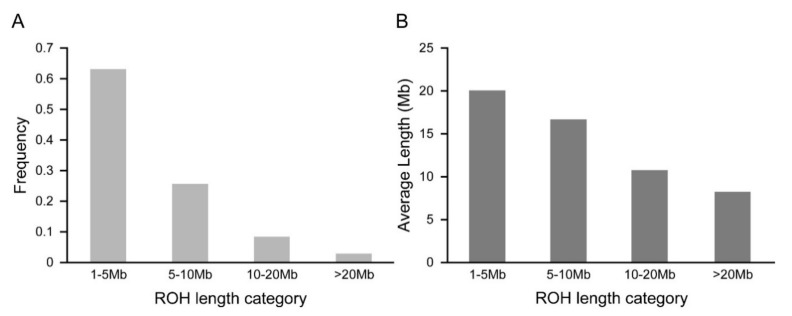
The percent frequency and mean sum of ROH in Chinese Merino under different length categories. (**A**) The percentage of ROH in the four ROH length categories. (**B**) The mean sum of ROH in the four ROH length categories.

The frequencies of ROH in Chinese Merino under different ROH length categories are presented in Figure 2A. The majority of detected ROH were less than 10 Mb (ROH 1–5 Mb and ROH 5–10 Mb), which accounted for 88.77% of the total number of ROH. Moreover, the frequency of ROH between 10 Mb and 20 Mb was 8.40%, while relatively few long ROH (more than 20 Mb in length) were detected. The mean genome coverages (in Mb) by ROH under different ROH length categories are shown in Figure 2B. The highest mean coverage of Merino genome was approximately 20.06 Mb in the 1–5 Mb ROH length group, whereas the lowest mean coverage was detected in the >20 Mb ROH length group.

**Figure 3:**
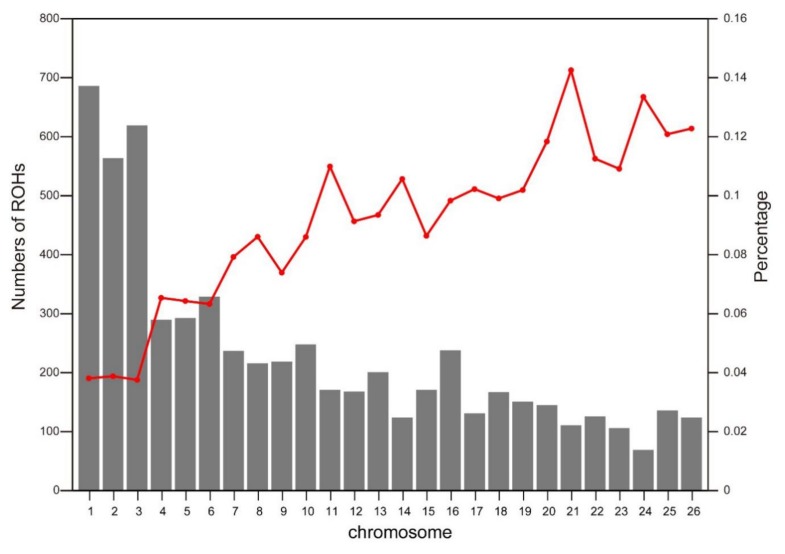
Total number of ROH per chromosome (gray bars) and average percentage of each chromosome covered by ROH (red line).

The number of ROH in each chromosome and the percentage of chromosomes covered by ROH are demonstrated in Figure 3. OAR1 displayed the highest number of ROH (*n* = 686), while the lowest number of ROH (*n* = 69) was observed on OAR24. The highest genome coverage by ROH was found on OAR21 (14.26%), while the lowest coverage was on OAR3 (3.76%). In overall, the number of ROH tended to reduce with decreasing chromosome length, and the percentages of genome coverage by ROH exhibited significantly different patterns.

Furthermore, the genetic position of each SNP was used to map the ROH hotspots, and the abundance levels of ROH in different length categories were used to assess the historical demographic patterns of the breed. The time to the most recent common ancestor (TMRCA) in the four different categories of the breed was evaluated (Figure S1). The power to deduce demographic patterns for >20 generations ago was limited due to the density of SNP array. A substantial increase in the abundance of ROH in Chinese Merino was observed from 10–20 generations ago to <5 generations ago, suggesting a recent decrease in the effective population size.

### 3.2. Inbreeding Coefficient Based on ROH

Descriptive statistics for ROH-based inbreeding coefficients (F_ROH_) under different length categories are presented in Table 1. The values of F_ROH_ gradually increased as a function of ROH length, ranging from 0.008 to 0.022. In addition, the coefficient of variation was also increased with increasing ROH length. As shown in Table 1, the mean F_ROH_ of Chinese Merino (0.023) was relatively low, with the highest F_ROH_ of up to 0.16.

**Figure 4:**
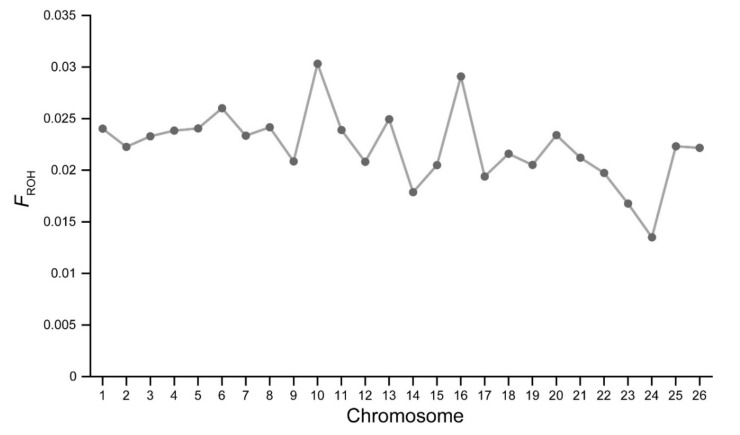
Distribution of ROH-based inbreeding coefficients (F_ROHOAR_) on each *Ovies aries* chromosome (OAR).

The distribution of F_ROH_ in each chromosome (F_ROHOAR_) is illustrated in Figure 4. Notably, the values of F_ROHOAR_ were comparatively different in the genome of Chinese Merino. The highest value of F_ROHOAR_ was found on OAR10, while the lowest value was observed on OAR24.

### 3.3. Genomic Regions with ROH Hotspots

**Figure 5:**
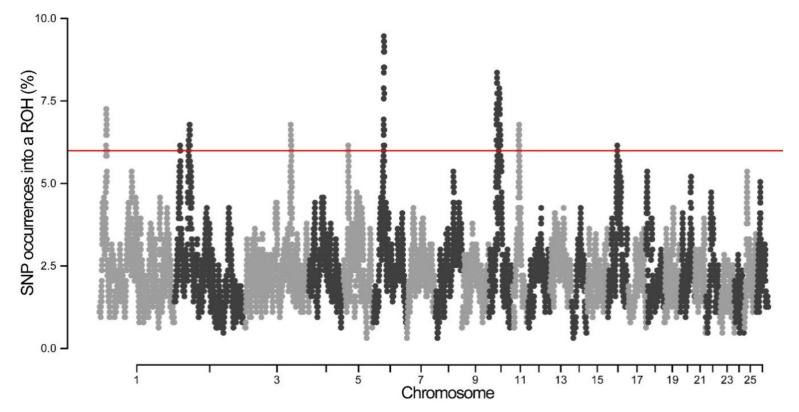
Manhattan plot of the occurrence of single nucleotide polymorphisms (SNPs) in ROH islands across different chromosomes.

By determining the abundance of SNPs in ROH, several genome regions were identified as potential ROH hotspots. As shown in Figure 5, the frequency of SNPs in ROH ranged from 6.15% to 9.46%. A total of 10 genomic regions on chromosomes 1, 2, 3, 5, 6, 10, and 11 were found to contain putative ROH hotspots (Figure 5, Table 2). The longest ROH island (7.31 Mb) was observed on OAR10, while the shortest one was observed on OAR11. The highest percentage of SNPs in ROH was found on OAR6, which consisted of 58 SNPs and 3.32 Mb ROH length (Figure 5, Table 2). To determine whether genetic recombination can affect ROH hotspots, the recombination rate of each hotspot was calculated based on the sheep genetic map. It was found that the recombination rates of ROH hotspots ranged from 0 to 1.32.

Furthermore, the identified ROH hotspots harbored 190 candidate genes (Supplementary Materials Table S1). Gene ontology (GO) and KEGG pathway analyses were carried out to determine the enriched functional genes (−log_10_^P^ > 2). The results indicated that the top 20 clusters were related to the regulation of cyclin-dependent protein serine/threonine kinase activity, positive regulation of immunoglobulin production, oocyte development, regulation of neuroblast proliferation, and regulation of growth and embryo implantation (Table 3).

## 4. Discussion

The length, frequency, and abundance of ROH constitute valuable sources of information on the historical demographic patterns of livestock species [25]. In this study, the characteristics of ROH in Chinese Merino were assessed using the Illumina Ovine 50K SNP BeadChip. The abundance of Chinese Merino was relatively similar to that of Australian Merino [26], but lower compared with some Italian sheep breeds [22] and commercial meat sheep breeds [19,26]. The lower ROH abundance of Merino sheep was consistent with their large effective population size [27,28]. There was a substantial increase in the abundance of ROH in Chinese Merino from 10–20 generations ago to <5 generations ago, suggesting a recent decrease in the effective population size in agreement with the formation history of Chinese Merino. The analysis of ROH length revealed that the majority of them were shorter than 10 Mb, which were consistent with the findings reported on the ROH length of sheep [19], pig [29], and cattle [6]. The length and number of ROH shorter than 5 Mb might probably be underestimated, as these ROH remained undetectable when using a medium-density SNP panel [6].

In Chinese Merino, the mean values of F_ROH_ were all less than 0.03, suggesting the low levels of inbreeding in this breed (Table 1). Similar F_ROH_ values were found in Italian sheep breeds and Spanish sheep breeds [21,22]. The main reason is that Chinese Merino sheep do not undergo intense selection and have a large effective population size [28]. The F_ROH_ values under different ROH length categories can be employed to reflect the present-day and ancient inbreeding [5]. According to the obtained F_ROH_ values (Table 1), the evidence of present-day inbreeding could become more overwhelming than that of ancient inbreeding. Furthermore, the maximum value of F_ROH_ (>20 Mb) was greater than 0.1, suggesting a relatively high level of relatedness among the individuals in this breed. However, these results consistently overlapped with the recent decrease in effective population, which might be attributed to the broad use of artificial insemination and decreased number of Chinese Merino raised in farm due to low wool prices in recent years.

In addition, ROH was used to measure the chromosomal inbreeding coefficients of Chinese Merino. Notably, the highest F_ROHOAR_ value was found on Oar10, and these findings were not consistent with those of the Valle del Belice breed [17]. Possible reasons for such difference may be due to the selection of different traits in sheep breeds. Selective breeding can reduce nucleotide diversity, increase linkage disequilibrium, and form a high level of homozygosity in genomic regions. Within OAR10, numerous quantitative trait loci associated with horn phenotype were detected, and the candidate gene related to horn development was also identified. Our previous works have indicated the high degree of linkage disequilibrium across OAR10 in Chinese Merino.

The maximum frequency of SNPs in ROH in Chinese Merino was around 6.15%–9.46%, Similar SNP frequency in ROH was found in Italian sheep breeds and Spanish sheep breeds [21,22]. On the contrary, the incidence of SNPs in commercial meat sheep breeds is higher than 20% [19]. In cattle, the ROH hotspots with the percentage of SNPs above 50% are more frequent than sheep [30]. These results reveal the hotspots in Merino and native sheep breeds are quite limited.

The existence of ROH hotspots might be partially attributed to genetic recombination rate [2]. The recombination rates within ROH hotspots ranged from 0 to 1.32 in Chinese Merino (Table 2). The previously reported recombination rates within ROH hotspots ranged from 0 to 0.87 [19] in commercial sheep and from 0.47 to 1.64 in Valle del Belice sheep [17]. The ROH hotspots in low recombination regions may be caused by selection process [19]. To verify whether these hotspots can overlap with putative selection signatures in sheep, we compared the hotspots region with previously reported selection signatures, and the results showed that five hotspots overlapped with a region under selection signature (Table 4).

The ROH hotspot with highest SNP occurrence was located on OAR6 (35.07–38.67 Mb), which consisted of the *NCAPG*/*LCORL* gene. Similar findings were also reported in Italian sheep breeds [22], Spanish sheep breeds [21], Swiss sheep breeds, and Merino [18,31]. Moreover, the selective signatures in *NCAPG/LCORL* region were found in many sheep breeds [18,32,33], including Merino [34]. A previous genome-wide association study (GWAS) demonstrated that this region was associated with body weight in Australian Merino [35]. In addition, this region was also associated with growth and body size in human [36,37], chicken [38], cattle, and horse [39,40,41]. Furthermore, the ROH hotspots on OAR11 from 28.02 Mb to 28.52 Mb and the corresponding selective signatures on the same gnome region were also reported by Kim et al. [42] and Signer-Hasler et al. [18]. A guilt-by-association study has also revealed that *TP53* is the most plausible functional candidate gene for body size in sheep [43]. Taken altogether, these results suggest that the ROH hotspots detected on OAR6 (35.07–38.67 Mb) and OAR11 (28.02–28.52 Mb) in Chinese Merino are caused by the artificial selection for body size trait performed on candidate genes.

The ROH on OAR10 (27.31–29.84 Mb) found in this study overlapped a common ROH island in Swiss sheep breeds [18] and Spanish sheep breeds [21]. More importantly, relaxin-like receptor 2 (*RXFP2*) gene was harbored in this genomic region. The ROH hotspot region also overlapped the selective signatures in *RXFP2* across many sheep breeds [27,32,45,46,47,48]. GWAS results demonstrated that *RXFP2* region was associated with horn phenotype in sheep [49]. The selective sweep region harbouring *RXFP2* was also identified in Merino [31], and this region was successfully used to predict the horned and polled phenotypes in Merino [50,51]. Additionally, the recombination rate on this genome region was relatively high. These findings support the effects of genetic recombination and selection on ROH hotspot formation.

Besides, the ROH hotspots on OAR2 (51.10–52.41 Mb) could be overlapped with a region under climate-associated selection that harbors two genes (e.g., *MELK* and *GNE*) [44]. The ROH hotspots on OAR5 (18.66–19.86 Mb) contained several immune-related genes (e.g., *IL4*, *IL13*, *IL5*, and *IRF1*) within the genome region, and this selection signature has been detected in sheep breeds [33].

## 5. Conclusions

In this study, the distribution patterns of ROH in Chinese Merino were analyzed based on the Ovine SNP50 BeadChip data. Our results demonstrated that the genome of Chinese Merino harbored a lower abundance of ROH, and the levels of ROH abundance were consistent with the effective population size. Moreover, both short and long ROH were detected, indicating that the ancient and recent inbreeding may have an impact on the genome of Merino sheep breed. It was also found that the ROH hotspots in sheep were formed through recombination and selection. In addition, several candidate genes were detected in ROH hotspots, revealing that the selection process for body size, horn, and immune traits in Chinese Merino has left distinctive signatures and formed ROH hotspots. The findings of this study can contribute to the identification of candidate genes associated with economically important traits in Merino sheep.

## Figures and Tables

**Table 1:** The number of genotyped animals and descriptive statistics of runs of homozygosity (ROH)-based inbreeding coefficient (F_ROH_) under different length categories.

Inbreeding Coefficient	Mean	Median	Minimum	Maximum	Coefficient of Variation	Number of Animals
F_ROH1-5Mb_	0.008	0.008	0.001	0.024	52.25	632
F_ROH5-10Mb_	0.008	0.006	0.002	0.031	69.30	541
F_ROH10-20Mb_	0.010	0.007	0.004	0.042	64.02	293
F_ROH>20Mb_	0.022	0.014	0.008	0.109	88.24	99
F_ROH>1Mb_	0.023	0.018	0.001	0.160	84.05	634

**Table 2:** ROH hotspots detected in Chinese Merino and average recombination rate (cM/Mb) within each hotspot.

No.	OAR	Start (bp)	Stop (bp)	Length (bp)	SNPs	Genes	cM/Mb
1	1	23294379	26901948	3607569	62	20	0.15
2	2	19930587	21080059	1149472	26	0	0.13
3	2	51106073	52409379	1303306	23	9	0.75
4	2	53415584	55758208	2342624	49	1	0.94
5	3	158391525	160963104	2571579	41	2	0.77
6	5	18659386	19857252	1197866	13	25	1.16
7	6	35075727	38668794	3593067	59	18	0.79
8	10	27305619	29842383	2536764	61	9	1.32
9	10	34325096	41802553	7477457	132	27	0.27
10	11	25425573	26939891	1514318	22	73	1.18
11	11	28019509	28518859	493350	14	5	0.96
12	11	28969704	28969704	0	1	1	/
13	16	32945561	32982579	37018	2	0	0

OAR = *Ovies aries* chromosome, SNPs = Number of SNPs in each ROH hotspot, Genes = Number of genes in each ROH hotspot.

**Table 3:** Gene ontology (GO) term and Kyoto Encyclopedia of Genes and Genomes (KEGG) pathway enriched data (−log_10_^P^ > 2) based on the annotated genes in ROH hotspots.

Category	Description	−log_10_^P^
GO Biological Processes		
GO:0000079	Regulation of cyclin-dependent protein serine/threonine kinase activity	3.84
GO:0002639	Positive regulation of immunoglobulin production	3.47
GO:0048599	Oocyte development	3.36
GO:0048839	Inner ear development	3.12
GO:0007568	Aging	3.03
GO:0060079	Excitatory postsynaptic potential	2.96
GO:0031214	Biomineral tissue development	2.76
GO:0036465	Synaptic vesicle recycling	2.69
GO:1902692	Regulation of neuroblast proliferation	2.68
GO:0047496	Vesicle transport along microtubule	2.24
GO:0043065	Positive regulation of apoptotic process	2.20
GO:0051052	Regulation of DNA metabolic process	2.20
GO:0040008	Regulation of growth	2.15
GO:0007566	Embryo implantation	2.14
**Reactome Gene Sets**		
R-HSA-5099900	WNT5A-dependent internalization of FZD4	3.71
R-HSA-190828	Gap junction trafficking	3.32
**Canonical Pathways**		
M219	PID hedgehog-GLI pathway	3.28
**KEGG Pathway**		
hsa04918	Thyroid hormone synthesis	2.58
hsa04961	Endocrine and other factor-regulated calcium reabsorption	2.24
hsa00330	Arginine and proline metabolism	2.16

**Table 4:** ROH hotspots detected in Chinese Merino overlap with the selection signatures in sheep.

OAR	Position (Mb)	Overlap Selection Signature Reference	Candidate Gene	Function
2	51.10–52.41	Lv Fenghua et al. [44]	*MELK, GNE*	Environment adaption
5	18.66–19.86	Naval-Sanchez et al. [33]	*IL4, IL13* *IL5, IRF1*	Immune function
6	35.08–38.67	Fariello et al. [32] Gutiérrez-Gil et al. [34] Naval-Sanchez et al. [33] Signer-Hasler et al. [18]	*NCAPG,* *LCORL*	Weight/height
10	27.31–29.84	Kijas et al. [27] Manunza et al. [45] Fariello et al. [32] Pan et al. [46] Kardos et al. [47] Randhawa et al. [48]	*RXFP2*	Horn
11	28.02–28.52	Signer-Hasler et al. [18] Kim et al. [42]	*FGF11, TP53*	Body size

OAR = *Ovies aries* chromosome.

## Supplementary Materials

The following are available online at https://www.mdpi.com/2076-2615/10/3/524/s1, Table S1: Candidate genes within ROH hotspots. Figure S1. The mean sum of runs of homozygosity (ROH) per animal estimated within four different generation categories. ROH were mapped according to their genetic positions. ROH length within each category was calculated using 100/2 g, replacing g with the number of generations of interest.
