# Supplementary material for: Genome-Wide Scan for Runs of Homozygosity Identifies Candidate Genes Related to Economically Important Traits in Chinese Merino
Source: Animals (Basel). 2020 Mar 20;10(3):524. doi: 10.3390/ani10030524 (PMC7143548; doi:10.3390/ani10030524)
Supplement: Supplementary file 1 [file animals-10-00524-s001.docx]

**Supplement Table 1.** Candidate genes within hotspots

| No. | Gene |
| --- | --- |
| 1 | ELAVL4, DMRTA2, FAF1, CDKN2C, C1orf185, RNF11, TTC39A, EPS15, NRDC, RAB3B, BTF3L4, ZFYVE9, CC2D1B, ORC1,  PRPF38A, TUT4, Vault, GPX7, SHISAL2A, COA7 |
| 2 |  |
| 3 | ZCCHC7, PAX5, MELK, RNF38, GNE, CLTA, CCIN, RECK, TMEM8B |
| 4 | TLE1 |
| 5 | SLC16A7, LRIG3 |
| 6 | GADD45B, LMNB2, TMPRSS9, SPPL2B, LSM7, LINGO3, PEAK3, OAZ1, DOT1L, SF3A2, AP3D1, IZUMO4, MOB3A, MKNK2, SEPTIN8,  CCNI2, KIF3A, IL4, IL13,IL5, IRF1, SLC22A5, SLC22A4, PDLIM4,  P4HA2 |
| 7 | GPRIN3, TIGD2, NAP1L5, HERC3, NAP1L5, HERC5, PPM1K, ABCG2, PKD2, SPP1, MEPE, IBSP, LAP3, MED28, FAM184B, NCAPG, DCAF16, LCORL |
| 8 | RFC3, STARD13, KL, PDS5B, N4BP2L1, BRCA2, ZAR1L, FRY, RXFP2 |
| 9 | MIPEP, TNFRSF19, SACS, SGCG, FGF9, MICU2, ZDHHC20, MRPL57, SKA3, SAP18, LATS2, XPO4, EEF1AKMT1, IFT88, CRYL1, GJB6, GJB2, GJA3, ZMYM2, ZMYM5, PSPC1, MPHOSPH8, PARP4, CENPJ, RNF17, ATP12A, PCDH9 |
| 10 | NLRP1, DERL2, DHX33, C1QBP, RPAIN, NUP88, RABEP1, SCIMP, ZFP3, KIF1C, INCA1, CAMTA2, SPAG7, ENO3, RNF167, SLC25A11, CHRNE, C17orf107, MINK1, PLD2, GLTPD2, VMO1, TM4SF5,  ZMYND15, CXCL16, MED11, ARRB2, PELP1, ALOX15, ALOX12,  RNASEK, BCL6B, SLC16A13, SLC16A11, CLEC10A, ASGR2,  ASGR1, DLG4, ACADVL, DVL2, PHF23, GABARAP, CTDNEP1, ELP5, CLDN7, SLC2A4, YBX2, GPS2, EIF5A, ACAP1, KCTD11,  TMEM95, TNK1, PLSCR3, NLGN2, SPEM1, C17orf74, TMEM102, FGF11, CHRNB1, ZBTB4, POLR2A, TNFSF12, TNFSF13, EIF4A1, CD68, MPDU1, SOX15, FXR2, SAT2, SHBG, ATP1B2, TP53 |
| 11 | NTN1, STX8, CFAP52, USP43, DHRS7C |
| 12 | DNAH9 |
| 13 |  |


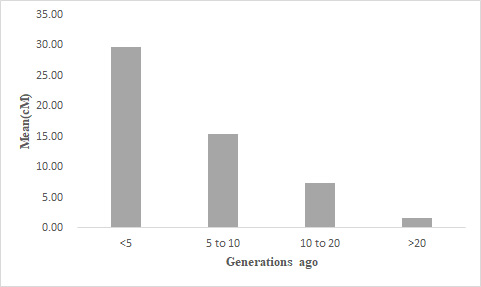


**Supplement Figure 1.** The mean sum of runs of homozygosity (ROH) per animal estimated within four different generation categories
